# Location-Aware Resource Discovery and QoR-Driven Resource Selection for Hybrid Web Environments

**DOI:** 10.3390/s21206835

**Published:** 2021-10-14

**Authors:** Lara Kallab, Richard Chbeir, Michael Mrissa

**Affiliations:** 1 Open Group, 92300 Levallois Perret, France; 2 Department of Computer Science, E2S UPPA, LIUPPA, University Pau & Pays Adour, 64600 Anglet, France; richard.chbeir@univ-pau.fr; 3 InnoRenew CoE, Livade 6, 6310 Izola, Slovenia; michael.mrissa@innorenew.eu; 4 Faculty of Mathematics, Natural Sciences and Information Technologies, University of Primorska, Glagoljaška 8, 6000 Koper, Slovenia

**Keywords:** hybrid Web environments, location-aware resource discovery, QoR-based selection, i-compositions

## Abstract

In the Web of Things (WoT) context, an increasing number of stationary and mobile objects provide functions as RESTful services, also called resources, that can be combined with other existing Web resources, to create value-added processes. However, nowadays resource discovery and selection are challenging, due to (1) the growing number of resources providing similar functions, making Quality of Resource (QoR) essential to select appropriate resources, (2) the transient nature of resource availability due to sporadic connectivity, and (3) the location changes of mobile objects in time. In this paper, we first present a location-aware resource discovery that relies on a 3-dimensional indexing schema, which considers object location for resource identification. Then, we present a QoR-driven resource selection approach that uses a Selection Strategy Adaptor (SSA) to form i-compositions (with i $\in N^{*}$) offering different implementation alternatives. The defined SSA allows forming resource compositions while considering QoR constraints and Inputs/Outputs matching of related resources, as well as resource availability and users different needs (e.g., optimal and optimistic compositions obtained using a scoring system). Analyses are made to evaluate our service quality model against existing ones, and experiments are conducted in different environments setups to study the performance of our solution.

## 1. Introduction

Nowadays, the REpresentational State Transfer (REST) architectural style [1] has become the most adopted solution for designing and developing Web services, also called RESTful services, i.e., self-contained and self-describing resources published to the Web. This is due to several advantages, among them, its simplicity, scalability, and support for different data formats, e.g., JSON (JavaScript Object Notation, http://json.org/) and XML (Extensible Markup Language, https://www.w3.org/standards/xml/core/). As the Web has become a major medium of communication [2], integrating objects (e.g., smart sensors) into the Web and taking advantage of its open popular standards, e.g., HTTP (Hypertext Transfer Protocol, https://tools.ietf.org/html/rfc7231/), has created a new concept: the Web of Things (WoT), which improves the interoperability and usability of the Internet of Things (IoT) [3]. In the WoT, objects can be (i) stationary (having invariant location), or (ii) mobile (their position changes over time), and are abstracted also as RESTful services (resources). Each resource is identifiable by a Uniform Resource Identifier (URI), and provides functions invokable using HTTP methods (e.g., GET, POST, PUT, and DELETE). A resource can be (i) dynamic, i.e., it may be connected to and removed from the Web environment at different instances, or (ii) static, i.e., it is established to be always connected to the environment. In this paper, a Web environment that allows one to connect static and dynamic resources is referred to as a “hybrid” environment.

There are many cases, in which, a single resource can not meet a specific client request, and often, answering some requests requires the combination of two or more resources, forming a composition that achieves the desired outputs. To form a composition, resource discovery [4] and resource selection [5] are performed. However, several challenges arise:

1. Identify WoT resources based on their object location: Objects, in mobile Web environments, may have variant or invariant locations. To collect relevant data from these objects, and provide pertinent results, it is important to consider their location to select their exposed resources that are the most appropriate for user demand. However, the huge number of objects that can be connected providing different resources functions, and their location changes in time, make the resource discovery process complex, especially when treating requests that need fast responses. Therefore, discovering resources, while considering their relative object location, in an effective manner and with acceptable delays, is necessary to satisfy user requests.
2. Select the appropriate resource for a needed function: Large Web environments can connect numerous candidate resources that provide the same function. With the huge number of candidate resources that may be connected, selecting the most relevant one, while taking into consideration user constraints (whenever they are given), becomes a complicated task for end-users. In this context, it is important to differentiate between the resources having the same function, to select the suitable resource for a function. This is done by considering Quality of Resource (QoR) attributes [6] (e.g., Availability and Cost). However, with the growing number of candidate resources having various QoR attributes [7], it is essential to have an automatic selection approach that can facilitate the task for end-users, and accelerate the process. In addition, and to provide compositions solutions that fit more efficiently the user’s demands, it is important to consider the matching of the input and output (I/O) of the related resources in a composition.
3. Form several composition alternatives: In hybrid Web environments, where dynamic resources can be connected, the selected dynamic resource(s) for a composition may be unavailable (e.g., disconnected from the environment) during the execution process. To prevent re-executing both the discovery and selection processes to create a new appropriate composition, it becomes essential to provide, during resource selection, i-compositions (i $\in N^{*}$), i.e., a set of compositions with different implementation alternatives. Such compositions achieve the workflow (which represents the dependencies between different functions to be satisfied by multiple resources) that is necessary to realize user demand by using, each, a different resource set. This gives the possibility to substitute a resource composition, in which some or all resources are no longer available (due to a disconnection from the environment for instance), by other compositions containing available resources. Thus, a selection approach that considers resource dynamicity is necessary. Also, in some cases, some users need optimal compositions having the highest scores, others may require optimistic compositions with acceptable scores but obtain with better delays, and in other cases, users ask for solutions having acceptable scores while considering resource dynamicity (whenever a dynamic resource is unavailable during a composition execution, there is always another composition consisting of available resources that can take over). Therefore, providing compositions solutions that answer different user requirements becomes important.

Web service discovery has received much attention in the literature [4,8,9,10,11,12]. However, none of the existing approaches are designed to handle both static and dynamic aspects of services. For example, works [4,8,9,10] focused on the discovery of services exposed by Web applications (i.e., static), without considering services dynamicity and mobility. Others [11,12] handled the discovery of mobile and dynamic services but neglected the existence of static ones. Also, many approaches addressed service selection [5,13,14,15,16]. Some works [5,13,15,17] considered Quality of Services (QoS) to select the most appropriate ones based on user constraints, without dealing with I/O matching between the related services and service dynamicity. Other approaches [16,18,19] handled the service selection problem as an AI planning problem to aim at finding a sequence of services that starts from some given inputs parameters and ends with the desired outputs parameters, with no consideration to services QoS and dynamicity. Moreover, and to the best of our knowledge, none of the existing composition approaches [20,21,22], is adaptive to provide various compositions types that answer different user demands (e.g., compositions with the highest scores, compositions with acceptable scores but formed with better delays, etc.).

To address the aforementioned challenges and existing limitations, we first present, in this paper, a location-aware resource discovery that relies on a 3-dimensional indexing schema for resource identification. The indexing schema, which maps the resources (static and dynamic), supporting HATEOAS (Hypermedia As The Engine Of Application State), which consists of including within resources responses, links to other resources, during their design, to identify the next possible resources to call based on the current resource state [1], to their functions and to the location of their relative objects (if they are exposed by objects), allows the identification of the data collection resources that are necessary to the required location relevant to the user request and enhances resource search in large Web environments. Then, we present a QoR-driven resource selection approach that uses a selection strategy adaptor that allows one to create different composition alternatives (i-compositions), while considering users QoR constraints, the matching between I/O parameters of related resources, as well as resource dynamicity and the composition type requested by the user. The proposed resource discovery and selection processes are automatic. This is done by using semantic annotations that are embedded into resources descriptions, which are expressed in this paper using Hydra [23] vocabulary.

In the following, we present our motivating scenario in Section 2, and present the handled main challenges and covered needs. Then, we discuss related work in Section 3. In Section 4, we detail our proposed resource discovery and selection approaches. Section 5 compares our defined QoR model against existing works and evaluates the performance of our solution. Finally, Section 6 concludes the paper and gives future directions.

## 2. Motivation, Challenges, and Needs

Our work targets hybrid Web-based environments that connect static resources, i.e., established to be always available on the Web, and/or dynamic resources, i.e., can be connected to and removed from the environment at different instants. The resources can be provided by Web applications or Web objects. There are many applications examples where our proposed resource discovery and selection approaches can be applied. As such, in the smart city context [24], we can found several use cases, e.g., Road Traffic Management, Smart Parking, Crowd-sensing, and Energy Efficient Buildings, which require huge data sensed from many connected devices, as well as preparing and processing the collected data using data preparation and analysis resources (services). The big number of data collection resources (exposed by mobile/stationary objects) that can be provided by such environments, the existence of numerous candidate resources having the same function, and the dynamic aspect of some of the resources, make resource discovery and selection challenging tasks to answer different user needs. The same challenges appear also in new application domains, such as Agriculture and Breeding [25], where there have been enormous changes in the technologies and methodologies for performing agricultural activities, among them, the incorporation of smart devices and the use of advanced processing data services for monitoring greenhouse conditions, smart farming, smart factory for reducing the maintenance cost, etc.

**Figure 1:**
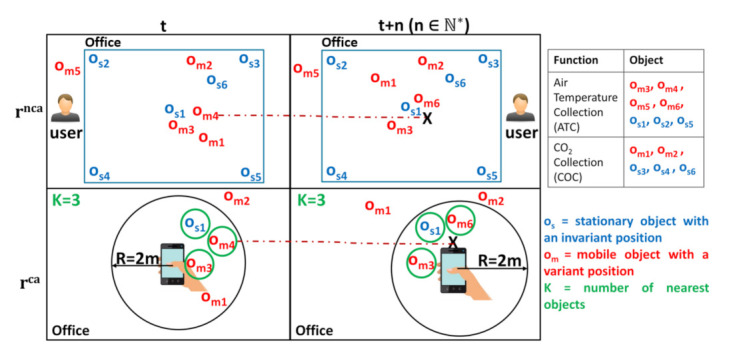
Examples of $r^{\mathrm{nca}}$ and $r^{\mathrm{ca}}$ in OpenCEMS.

Although the different application examples, in this paper, we consider the following scenario that is related to a Web platform called “OpenCEMS” (Connected Environment & Distributed Energy Data Management Solutions: https://opencems.sigappfr.org/). OpenCEMS, which is currently under development, offers several solutions to manage the energy data in connected environments (e.g., smart buildings, cities, and factories). It can connect (1) objects (with variant/invariant location) providing static or dynamic resources, and (2) Web applications, which are established to be always available in OpenCEMS, and thus, published as static resources. The resources, described in Hydra and registered in a triplestore, are mainly used for: collecting heterogeneous on-site data, preprocessing collected data, and analyzing data. In our scenario, a building manager desires to predict his office temperature, to check if the installed temperature-controlled system works properly. His demand, which is expressed using the ATP (Air Temperature Prediction) function, can be sent via 2 requests types: (1) $r^{\mathrm{nca}}$, which refers to a non-context aware request, where the necessary data for his demand is collected using objects (devices) that are in his office, without considering his position, and (2) $r^{\mathrm{ca}}$, a context-aware request, in which the required data for his demand is provided by devices dependant from where he is standing in his office (e.g., from the 3 nearest devices located in his office with a 2m range). To satisfy the building manager’s request, it is important to identify the required resources and select the appropriate ones to answer his demand. Nevertheless, as illustrated in Figure 1, the following challenges emerge:

1. Discover the suitable resources that collect the required data. To have accurate data for the building manager demand, identifying the resources that are (i) located in his office, or (ii) the nearest to his standing point in his office, is important. For instance, object $o_{m5}$ provides a resource offering the ATC (Air Temperature Collection) function, which is necessary to collect the necessary air temperature data, but it is not located in the office of the building manager. Therefore, the provided resource would be unuseful for the request $r^{\mathrm{nca}}$ at instants t and t + n. However, at instant t, there are 5 objects ($o_{s1}$, $o_{s2}$, $o_{s5}$, $o_{m3}$, and $o_{m4}$) satisfying ATC that are located in the building manager office and can answer $r^{\mathrm{nca}}$ more efficiently. As for $r^{\mathrm{ca}}$, i.e., Range-KNN type [26], at instant t, $o_{s1}$, $o_{m3}$, and $o_{m4}$ are the most appropriate to use (as they are the nearest to the building manager standing point and within the required 2m range), while at t + n, $o_{s1}$, $o_{m3}$, and $o_{m6}$ are more convenient. Nevertheless, the existence of numerous mobile data collection resources, makes their discovery a time-consuming task. Thus, finding suitable resources in a huge Web environment with an acceptable response time is important to answer user requests efficiently.
2. Select the necessary resources forming an appropriate composition. With the existence of several candidate resources providing identical required functions for the building manager request, such as ATC, selecting the most interesting one is a tedious task to do, as it requires time and expertise. Therefore, and to facilitate such task, QoR are used to differentiate between the candidate resources and to help in choosing the appropriate ones. For instance, object $o_{s1}$ can be better than the others as it may have: (i) a high capacity of the battery, which denotes a high availability, (ii) a stable connection to the attached Web environment (as it is static), (iii) no cost when executing it, and (iv) a better usage rate comparing to other (as it may have been used several times in other scenarios). Considering the QoR, i.e., Availability, Dynamicity, Cost, and Usage in this work, allows one to select the suitable resources among all other candidates. Since numerous resources providing the same function can be offered by OpenCEMS with different QoR values, an automatic resource selection solution is essential to ease the selection task and make it faster. In addition, and to ensure composition results that are more efficient to user demands, the solution should consider the matching between the I/O parameters of the related resources forming a composition.
  Moreover, in some cases, the building manager may require:
  (a) Results provided by the qualified resources among all others. In such case, the resources that are to be selected are those with the highest quality aspects values among others. This is done independently of the response time of the selection process and may be useful in many cases as in when the building manager requires, for instance, to regulate the temperature of his office for a business meeting that will be held the next day.
  (b) Fast but acceptable results. As the building manager may feel very hot where he stands in his office, he requires fast predicted results to adjust the ambient temperature. To do so, it is important to select suitable resources that can answer his request without checking all the others. Despite demanding fast predictions, it is essential to ensure efficient results. This can be done by selecting resources that have acceptable (minimal) quality aspects values.
  (c) Results obtained at any time. In such case, the building manager is required to receive predicted results at any time of demand, even though some selected dynamic resources may not be available anymore (disconnected from OpenCEMS for instance). To do so, it is important to find resources that are always available to satisfy the building manager demand at any instant.
  In addition, and for each of these previous requirements, the building manager may have other particular needs, such as:
  - Results obtained with no cost, which can be obtained by selecting resources having zero cost.
  - Results that are effective, which can be acquired by selecting resources having a high rate of availability.
  - Results that are efficient, which is done by selecting resources having a high usage rate, i.e., they have been used many times before in other different scenarios. As such, the more a service is called to answer different user demands, the more it proves its efficiency in various scenarios.
  - Results that are reliable, which can be obtained by selecting resources that are can be linked properly together by considering their I/O parameters matching.
  To satisfy different user needs, it is necessary to consider his constraints and adapt resource selection accordingly.
3. Form multiple resource compositions alternatives. Due to their dynamic aspects, some dynamic resources that are selected in a composition cannot be available anymore for execution. For example, at instant t and for $r^{\mathrm{nca}}$, 5 objects (having a variant position) are located in the office of the building manager, and expose dynamic resources offering the ATC function. If we consider that object $o_{m4}$ provides the most suitable resource among all the other objects, as it may have the highest qualify aspects values, for instance, it shall be selected to be in the necessary composition to answer the building manager request. However, at any instant (at t + n for example), $o_{m4}$ can be unavailable (disconnected from OpenCEMS). At this point, the composition will no longer be efficient when executing it after ⩾t + n, as it misses a selected resource providing a required function. The same scenario happens to $r^{\mathrm{ca}}$ in which $o_{m4}$ is not available anymore. To prevent re-executing the resource discovery and the resource selection processes, and create another appropriate composition solution formed by available resources, identifying several composition alternatives (i-compositions with i $\in N^{*}$) during resource selection becomes important.

In the following section, we present the most interesting approaches related to our scope of work (resource discovery and selection) and evaluate them according to the aforementioned challenges and needs.

## 3. Related Work

### 3.1. Discovery Approaches

In the Web service domain, several works addressed service discovery. In [27], a solution is defined for the discovery of static resources supporting HATEOAS and connected WoT dynamic resources. The solution models the resources in a single resource graph, and adapts graph algorithms to explore resource descriptions, to identify the resources answering a user request. In [8], a resource discovery approach based on the Breadth-First Search algorithm is proposed. It explores semantically annotated resources descriptions to identify the resources realizing the required functions. In [9], a Web service description and interaction approach for automatic Web service discovery is proposed. It is based on Notation3 Resource Description Framework syntax to describe REST services and uses operational semantics of Notation3 to allow a flexible discovery. In [4], a service description model is defined to generate a graph capturing state transitions in an activity layer, resources, transitions, and response semantics in a semantic layer. Using graph queries, the graph is traversed to discover the services. The work in [10] proposes a solution for automatic discovery and consumption of data sources at Web scale. The solution is based on a SPARQL (a query language for RDF format, https://www.w3.org/TR/rdf-sparql-query/) Service Description (SD) document to describe micro-services. The SD document is linked to a SHACL shapes graph (https://www.w3.org/TR/shacl/) that describes the resources manipulated by the micro-service, and to other vocabularies as Hydra to have a richer functional description. The SD can be also dynamically transformed into a Web page enabling its discovery using common Web crawlers.

Resource discovery is also an active research area in other domains. As such, in the Internet of Things [11] and Mobile Cloud Computing [12] solutions mainly allow the discovery of resources based on their location and context properties (e.g., Accuracy and Precision). Other works related to agents and workflow scheduling also considered service discovery. In [28], agents and services are assembled into a multi-agent framework called Agent-Oriented Resource mAnagement (AGORA). Each AGORA provides specific services, and several AGORAs can be combined forming a graph. Service discovery begins in the requested AGORA. If the latter does not support the required services, the other linked AGORAs are used. As for workflow scheduling-based solutions [29,30], they consist of mapping the required workflow tasks to the most existing suitable resources while considering user constraints.

**Discussion:** Although the work [27] discovers static and dynamic resources, it does not consider objects location during resource identification. The works [4,8,9,10], focus on the discovery of resources that are exposed by Web applications, and not WoT objects, thus, neglecting the mobility and dynamicity aspect of resources. In the works [11,12] focus more on the resources exposed by connected objects, without considering their possible relations with the resources exposed by Web applications (i.e., static). In [28], where resource discovery is done through traversing an AGORA-based graph instead of a resource-based graph, resource dynamicity and location are not handled. As for [29,30], they do not consider resources dynamicity and location.

### 3.2. Selection Approaches

#### 3.2.1. QoS-Based Approaches

Authors in [5] present a quality-driven resource selection approach, in which each resource is described using Hydra, and several quality aspects of resources are embedded into the hydra-based resources descriptions. The solution uses a skyline-based algorithm to reduce the number of candidates resources having identical task. In [14], a QoS-aware Web service composition problem is solved by proposing a heuristic. In the approach, a backtracking algorithm is applied to the results calculated using a Linear Program relaxation, and user constraints, which are defined to the overall service composition, are considered. The work in [15] presents a service selection process that considers qualitative and quantitative user QoS preferences, as well as services trust properties. The proposed solution can be used in Big Data Web-based environments, which include a huge number of services (i.e., business applications) that are migrated to the cloud. In [31], a QoS-aware service composition is proposed based on QoS correlations. The solution allows one to generate the optimal composite service by considering service plans along with sequence structure. The work in [13] presents an enhanced algorithm based on Artificial Bee Colony (ABC) to deal with the QoS-aware Web service selection process problem. The enhancement is based on a two steps process: using neighboring nodes to enhance the performance of the ABC algorithm by encouraging exploration in early iterations, and randomly swapping portions among the best two solutions randomly to exploit the characteristics of the best solutions to generate new solutions.

**Discussion:** The aforementioned works use QoS aspects while selecting services, and most of them consider user constraints/preferences. However, the services dynamicity aspect, and I/O parameters matching between the related services, are neglected. In addition, none of the solutions allows the generation of different service composition types that can answer the different user’s demands.

#### 3.2.2. I/O-Based Approaches

In [16], a graph-based framework to automate service composition is proposed. The framework can generate a service composition consisting of minimum services number. This is done by focusing on the semantic matching of the I/O parameters services, and thus, several compositions can be retrieved to answer user demand expressed using inputs and outputs terms. The work in [18] provides a formal model for an AI planning-oriented service composition. In the solution, the I/O semantic similarity between the services is calculated based on causal links. The latter are logical dependencies between the inputs and outputs parameters of the different services. Authors in [19] propose a method that increases the expressiveness of the Web services parameters, by describing them with properties and defining binary relations (inheritance) between them. The work describes how Web service parameters, whose properties can be inherited, are used to form, automatically, valid compositions.

**Discussion:** All of the aforementioned works consider I/O services semantics to select the services for a composition. However, this is done without taking into account the functions provided by the possible linked services. In addition, the solutions neglect the services dynamicity aspect, as well as QoS aspects and user constraints or preferences.

#### 3.2.3. K-Compositions Approaches

Authors in [20] present an automatic service composition approach to retrieve the top-k compositions. For this matter, the work applies the MapReduce concept, by mapping, into several tasks, the top-k compositions which can be run in parallel. In the solution, only one QoS metric, i.e., Response time, is used, and similarities between I/O services are computed for services filtering. The work in [21] proposes an approach that allows one to compose the top-k DaaS (Data as a Service) services. The formed top-k service compositions are produced using a fuzzy score that is associated with every service and service composition, along with the given fuzzy user preferences, which are defined by fuzzy terms (e.g., “cheap" for services price). Authors in [22] resolve the QoS-aware service composition problem through a relational database. The proposed solution generates all possible service combinations and stores them in a relational database. Based on a given user request, SQL queries are composed to search in the database and return the top-K Web service compositions having the best QoS.

**Discussion:** All of the aforementioned works generate multiple service compositions and take into consideration QoS aspects. Nevertheless, they do not deal with service dynamicity, and they do not allow the generation of various composition services types to satisfy different user demands.

## 4. Location-Aware Resource Discovery and QoR-driven Resource Selection for i-Compositions

### 4.1. General Overview

**Figure 2:**
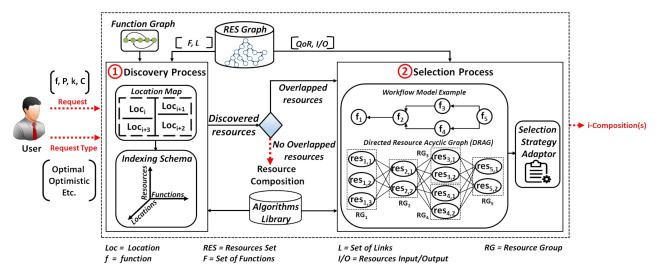
Overview of the resource discovery and selection processes.

Figure 2 shows an overview of our resource discovery and selection processes, applicable in hybrid Web environments providing static and/or dynamic resources.

Based on user request and user request type, the two processes allow forming i-compositions satisfying user needs. The user request type can hold 3 main different composition types to be formed:

- Optimal, which refers to resource compositions with the highest scores.
- Optimistic, which refers to resource compositions with minimally acceptable scores (see Section 4.4.2), and obtained in satisfactory delays.
- Hybrid, referring to resource compositions with acceptable scores, but in which, at any instant of the Web environment runtime, the existence of a resource composition is ensured, by considering the dynamicity aspect of resources.

Optionally, the main composition types may be followed by other composition subtypes (e.g., trusted, which refers to resource compositions that consist only of static resources, and cost-free, which denotes resource compositions that consist of resources with no charge). Besides specifying the composition types and subtypes, the user defines his demand through a spatial request, r, presented in our work to be applied in WoT-based environments, where objects (e.g., smart devices) can be connected. r can be (1) context aware request, $r^{\mathrm{ca}}$, in which the location of the requesting user device is considered during the processing, or (2) non-context aware request, $r^{\mathrm{nca}}$, where the position of the requesting user device is not included into the process.

In this work, $r^{\mathrm{ca}}$ = $r_{\mathrm{range}}^{\mathrm{ca}}\left|r_{\mathrm{knr}}^{\mathrm{ca}}\right|r_{\mathrm{rknr}}^{\mathrm{ca}}$, with: $r_{\mathrm{range}}^{\mathrm{ca}}$ is a range request type [32], $r_{\mathrm{knr}}^{\mathrm{ca}}$ is a K Nearest Resource (KNR) request type [33], where the nearest resources are provided by the nearest objects to the requesting device location, and $r_{\mathrm{rknr}}^{\mathrm{ca}}$ is a Range-K Nearest Resource (R-KNR) request type [26]. More formally, we define r as:

**Definition** **1.** ***r*** *= (f,P,k,C), where:*

- ***f*** *, is the user requested function, selected from a generated list of functions, F, that can be provided by the resources connected to the Web environment at the current instant. F ∈ FG, where FG refers to the directed acyclic function graph that defines the order dependencies of F.*
- ***P*** , refers to the parameters set required to execute f, such that P = ${⋃}_{i=1}^{N^{*}}\left\{p_{i}\right\}$, where $p_{i}$ = (key:value), and with: key, denoting the name of the parameter, and value, referring to the user given value of the parameter. We define in P: (i) a location parameter, Location:value, where value denotes the required location (e.g., office), and (ii) a scope parameter, Scope:value, with a value representing a range $\in R^{+}$. Using the location and the scope parameters, the user can define if the processing of his request considers data collected from objects that are: (1) positioned in a given location, or (2) positioned in a specified location and covering a given scope, or (3) located in a scope limited by a circle with a specific radius and whose center is the location of the user requesting device.
- ***k*** *∈N, refers to the number of objects used to collect data and that is the nearest to user location at request time. k also refers to the number of the necessary resources to be discovered that provide functions other than collecting data. When k = 0, all of the resources providing the necessary functions to fulfill f, as it is defined in FG, are to be discovered.*
- ***C**, is the given user constraints according to which, i-compositions are obtained.* ***C*** = $Q_{c}\cup i\cup W\cup d$ *, with:*
  ○ **
$Q_{c}$** *= $Q_{c}^{\mathrm{res}}\cup Q_{c}^{f}$, refers to the set of constraints given to the resources ($Q_{c}^{\mathrm{res}}$) and to their provided functions ($Q_{c}^{f}$), with $Q_{c}^{\mathrm{res}}$ = ${⋃}_{i=1}^{n}\left\{q_{i}^{\mathrm{res}}\right\}$, and $Q_{c}^{f}$ = ${⋃}_{j=1}^{m}\left\{q_{j}^{f}\right\}$, and where:*
    - *n denotes the number of attributes describing a resource (we use “Dynamicity” and “Availability” in this paper), and m refers to the number of attributes used to describe the resource provided functions (we use “Cost” and “Usage” in this work).*
    - *$q_{i}^{\mathrm{res}}|q_{j}^{f}$ = [$\min_{i|j}$-$\max_{i|j}$], where $\min_{i|j}$, $\max_{i|j}$ denotes, respectively, the minimum values and maximum values defined by the user for $q_{i}^{\mathrm{res}}$ and $q_{j}^{f}$.*
  ○ ***i*** $\in N^{*}$, refers to the user desired compositions number. By default, it is equal to 1 and can be specified only for the resource compositions that are either optimal or optimistic. As for the hybrid resource compositions, their number depends on the dynamicity aspect of the resources (see Section 4.4.2).
  ○ ***W*** *= $\left\{w_{\mathrm{qor}},w_{\mathrm{io}}\right\}$, are the weight values given respectively to the score sum of the resources in a composition (based on their QoR attributes) and to their I/O matching, while computing compositions score (see Section 4.4). $w_{\mathrm{qor}}$, $w_{\mathrm{io}}$$\in R^{+}$ and are bounded by [0, 1]. By default, W = $\left\{1,1\right\}$.*
  ○ ***d*** *, refers to the value rate degree (expressed in %) of a calculated threshold, T (see Section 4.4.2), which denotes the acceptable minimal score of the optimistic and hybrid i-compositions.*

As illustrated in Figure 2, our solution consists mainly of (1) the Discovery Process (DP), and (2) the Selection Process (SP). First, when DP receives the user request, r, it uses a 3-dimensional indexing schema that consists of 3 dimensions: Function, Resource, and Location, to identify: (i) the necessary resources that provide data collection functions (whenever required for r) in the needed location (i.e., defined in a location map), or (ii) the necessary resource(s) that will be used by DP to crawl the Web graph of resources (whenever there are no data collection functions that are needed). The traversal of the Web resources graph is done by using a graph-based algorithm, e.g., Breadth-First Search (BFS) and Depth First Search (DFS) [34], from the algorithms library. With respect to the HATEOAS principle in REST, which consists of linking the resources together, the Web resource graph, denoted in our work as RES graph, is formed by the hypermedia resources links embedded in resources descriptions (expressed with Hydra in this work). This allows DP to dynamically navigate to the next resources and discover the ones providing the functions required for r. Such functions form a Workflow Model (WM) that is defined in the function graph (FG). When there are no candidate resources that are discovered for the required functions in WM, only one composition is returned. If there are candidate resources for at least one required function, SP is executed.

In the selection process, SP, the resources having a similar function are grouped into the same resource group, RG. Each resource in an RG can be linked to the resources included in the RG related to the next function, as defined in the Workflow Model (WM), forming a Directed Resource Acyclic Graph, DRAG. DRAG is traversed by a Selection Strategy Adapter (SSA) that adapts to user needs (the user request and the user request type) to form the required i-set of resource compositions. SSA allows producing optimal resource compositions with the highest scores, and resource compositions with minimally acceptable scores, i.e., ⩾a computed threshold, T, obtained with better delays. T is calculated and used for the optimistic and hybrid composition types.

### 4.2. Preliminaries

In our work, a resource, res, can be static, ${\mathrm{res}}^{s}$, or dynamic, ${\mathrm{res}}^{d}$, and is formally defined as:

**Definition** **2.** ***res*** **= (c,id,loc,F,L,** *$Q_{\mathrm{res}})$:*

- ***c*** *, is the Web address of the context containing terms that are linked to existing data models (e.g., ontologies). These terms map the properties of the resources to concepts that are defined in their relevant data models.*
- ***id*** *, refers to the Web address (URI) of the resource res.*
- ***loc*** *, denotes the location of the object that exposes the resource res (whenever it is the case).*
- ***F =*** ${⋃}_{i=1}^{N^{*}}\left\{f_{i}\right\}$, refers to the set of the functions that are provided by the resource res, where: $f_{i}$ = ($n,I,O,m,Q_{f}$), and with:
  ○ ***n*** *denotes the name of the function $f_{i}$.*
  ○ ***I*** *refers to the the input(s) of the function $f_{i}$.*
  ○ ***O*** *refers to the the output(s) of the function $f_{i}$.*
  ○ ***m*** *denotes the HTTP verb that is used to invoke the function $f_{i}$.*
  ○ $Q_{f}$ = ${⋃}_{i=1}^{N^{*}}\left\{({\mathrm{qf}}_{i}:{\mathrm{vf}}_{i})\right\}$, refers to the quality attributes set related to the function $f_{i}$, with ${\mathrm{qf}}_{i}$ denoting the attribute name (Cost and Usage in this paper), and ${\mathrm{vf}}_{i}$ *∈* $R^{+}$.
- ***L*** *, the set of links (if they exist) to other resources. ${\mathrm{res}}^{s}$ can be directly linked to another ${\mathrm{res}}^{s}$, however, the linking between ${\mathrm{res}}^{s}$ and ${\mathrm{res}}^{d}$ is done using virtual resources, similar to the work in [27].*
- $Q_{\mathrm{res}}$ = ${⋃}_{i=1}^{N^{*}}\left\{({\mathrm{qres}}_{i}:{\mathrm{vres}}_{i})\right\}$, the set of quality attributes related to res, with ${\mathrm{qres}}_{i}$ is the name of the attribute (Dynamicity and Availability in this work), and ${\mathrm{vres}}_{i}$∈$R^{+}$.

In the literature, there are multiple QoR attributes, e.g., Availability and Cost, that are used to differentiate between the candidate resources providing similar functions [6]. In this work, some attributes are related to the resources themselves ($Q_{\mathrm{res}}$), and some others are related to the resources provided functions ($Q_{f}$):

- **Dynamicity**, is the quality attribute that denotes whether a resource is established to be available, i.e., a static resource (${\mathrm{res}}^{s}$), or not, i.e., dynamic resource (${\mathrm{res}}^{d}$). Dynamicity = 0, if the resource is static, and Dynamicity = 1, if the resource is dynamic. Users in their request, r, can specify whether they require dynamic and/or static resources while forming the i-compositions. In this context, and for the values that are given to the Dynamicity attribute ($q_{1}^{\mathrm{res}}$) in $Q_{c}^{\mathrm{res}}$, when $q_{1}^{\mathrm{res}}$ is:
  ○ [1-0], only the static resources can be part of the necessary compositions.
  ○ [1-1], both static and dynamic resources can be used to form the necessary compositions.
  ○ [0-1], dynamic resources can only be part of the necessary compositions.
- **Availability**, refers to the degree (expressed in %) to which a resource res is operational or is ready for immediate use. For the resources that are exposed by connected objects (e.g., devices), the Availability attribute refers to their battery capacity.
- **Cost**, denotes the amount of money that is required to be paid (in a specific currency) to use a resource function. The Cost attribute can be either defined by the provider of the resource (i.e., the person or organization that developed/created the resource) or by the provider of the object exposing the resource (i.e., the one that is connecting the object to the Web environment).
- **Usage**, refers to a value that increases when a function of a resource is used. By default, the Usage attribute is equal to 0. To prevent the re-initialization of the Usage value every time a dynamic resource, ${\mathrm{res}}^{d}$, is disconnected, for instance, we define for each function of a dynamic resource, a Time To Live (TTL) value that denotes the maximum time during which a dynamic resource can be unavailable before decreasing the Usage value by 1.

**Figure 3:**
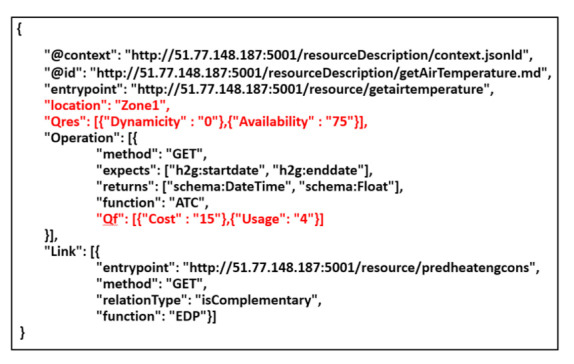
Example of an extended Hydra-based resource description.

Based on res definition, we extended Hydra-based resource description to additionally include: (i) the location of a resource exposed by an object (See Section 4.3), and (ii) the QoR values. An example of an extended Hydra resource description is shown in Figure 3.

QoR attributes are categorized as (i) maximization attributes, which are to be maximized (e.g., Availability), and (ii) minimization attributes, which are to be minimized (e.g., Cost). The QoR attributes are used for computing, for each provided function of a resource, a global score defined as: score(${\mathrm{res}}_{f}$) = $\sum_{i=1}^{N^{*}}\left\{{\mathrm{vres}}_{i}\right\}+\sum_{i=1}^{N^{*}}\left\{{\mathrm{vf}}_{i}\right\}$, where ${\mathrm{vres}}_{i}$ (excepting the Dynamicity attribute) and ${\mathrm{vf}}_{i}$ are normalized based on the Equation (1) or (2) presented below. As such, and due to the QoR different units and dimensions, it is essential to normalize their values while computing the score(${\mathrm{res}}_{f}$). Similar to [35], Equations (1) and (2) are used to normalize the attribute values of group (i) and group (ii) respectively, with $q_{i}$ = ${\mathrm{vres}}_{i}|{\mathrm{vf}}_{i}$, denoting the attribute value that is related to a resource, or the attribute value corresponding to a resource provided function. In the equation below, a normalised attribute value, $q_{i}^{\prime }$, is equal to 1 if $\max\left(q_{i}\right)-\min\left(q_{i}\right)$ = 0, with $\max(q_{i})$ and $\min(q_{i})$ refers respectively to the maximum and minimum values of $q_{i}$ among the identified resources in the Directed Resource Acyclic Graph (DRAG):

(1) $$q_{i}^{\prime }=\frac{q_{i}-\min\left(q_{i}\right)}{\max\left(q_{i}\right)-\min\left(q_{i}\right)}$$

(2) $$q_{i}^{\prime }=\frac{\max\left(q_{i}\right)-q_{i}}{\max\left(q_{i}\right)-\min\left(q_{i}\right)}$$

### 4.3. Location-Aware Resource Discovery

**Figure 4:**
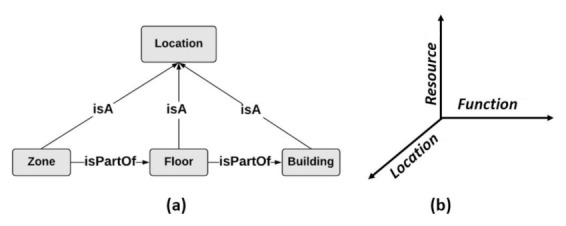
An example of a geographic hierarchy VS The indexing schema.

To identify the suitable resources used for collecting data, and which are relevant to the specified location in the user request, r, we consider, in this work, that there is a location map that describes the geographic area related to the Web environment. Such a location map contains different location levels defined in a specific geographic hierarchy. As an example, in the motivating scenario, presented in Section 2, and which is related to connected environments, i.e., smart buildings in our case, we assume that Zone $\overset{\mathrm{isPartOf}}{⟶}$ Floor $\overset{\mathrm{isPartOf}}{⟶}$ Building, where Zone, Floor, and Building are entities that refer to location types (subclasses of “Location”), as it is shown in Figure 4a. Zone is the smallest location granularity, such that Zone = $Z_{i}$, with i ∈N. In this paper, we consider that the location of an object is periodically updated according to a defined time interval. To discover the resources realizing r, and locate the suitable data collection objects with acceptable response time, we define an indexing schema, IdS, that consists of 3 dimensions: Function, Resource, and Location, as presented in Figure 4b. IdS is formally defined as

**Definition** **3.** ***IdS = (F, R, L*** *):*

- ***F*** *= $\left\{x\right\}$, refers to the x-axis that holds abscissa values denoting: (i) the indices of the functions provided by the static resources, and (ii) the data collection functions offered by the dynamic resources. Each x value has a “fsignature" that consists of the indices of the functions that are necessary to realize f, as it is defined in FG.*
- ***R*** *= $\left\{y\right\}$, refers to the y-axis that holds ordinate values denoting the set of all of the static resources and the dynamic resources that provide functions for collecting data. Each y value referring to a static resource, ${\mathrm{res}}^{s}$, has a “rsignature" which consists of the indices of the resources that are related to it through the semantic relations “isSimilar" and “isComplementary" [27]. As dynamic resources can be disconnected at different instants from the Web environment, they do not have any defined related resources. Thus, signatures are not specified for dynamic resources.*
- ***L*** *= $\left\{z\right\}$, refers to the z-axis that holds the applicate values representing the set of the smallest location granularity (zone for example) of the connected objects providing static or dynamic resources used for data collection.*

Using IdS, the resource discovery process, described in Algorithm 1, can identify the resources providing terminal functions that are independent of any other (data collection functions in this work), and relative to the necessary location, in an efficient manner.

Algorithm 1 presents the resource discovery process having the following input data:

− **algoType** (string): denotes the algorithm type to be used (i.e., BFS or DFS in this work), that is adapted to traverse RES graph formed by the linked resources following the HATEOAS principle.
− **f** (string): is the user requested function.
− **P** (array of [string, string]): is the set of the values of the parameters relative to the location and to the scope specified by the user for data collection objects.
− **k** (integer): is the maximum number of the discovered resources providing identical functions.

**Algorithm 1:** The Discovery Process (DP)
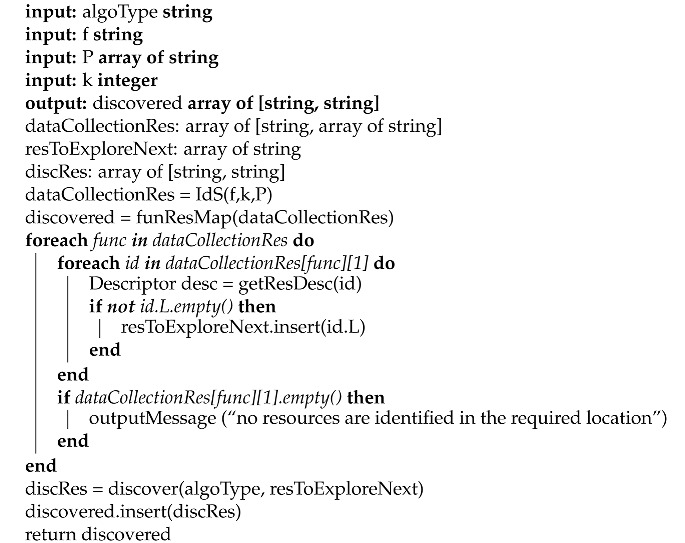


The algorithm output is the **discovered** array, which contains the pairs [**f**, **id**] corresponding to the identified resources that are necessary to provide f. The process of resource discovery consists of the following main functions:

- **IdS (string, integer, array of [string, string])**, is used to identify the k number of the resources that provide the data collection functions necessary to realize f, and which are relevant to the location specified by the user, using the indexing schema.
- **funResMap (array of [string, array of string])**, is used to produce an array of [string, string] that maps each connected resource to its corresponding function.
- **getResDesc (string)**, is used to get the descriptions of the resources, each identified by its own id (i.e., URI), and the set of the related resources, which can be traversed next.
- **discover (string, array of string)**, is used to traverse the RES graph. This is done by starting from the set of the identified resources from the indexing schema and passing by their related resources that are necessary to realize f. Its algorithm is presented in Appendix A.

The discovery process (DP) begins by discovering the resources that provide the required data collection functions for the user requested function (f), and which are positioned in the necessary location, by calling the **IdS(f,k,P)** function (line 9). The set of the required functions used for collecting data along with their corresponding discovered resources are saved in the **dataCollectionRes** array. The **funResMap(dataCollectionRes)** function is then used to map every function to its relevant resource, and stores the outputs in the **discovered** array (line 10). For each resource in **dataCollectionRes**, DP gets its relevant resource description (which is expressed in Hydra vocabulary in this work) by calling the **getResDesc(id)** function (line 13), to retrieve their linked resources ids that are to be traversed next. These resources ids, that are stored in the **resToExploreNext** array (line 15), are used later by the **discover(algoType, resToExploreNext)** function, which is presented in Appendix A, to discover the resources providing the required other functions. If no resource is identified providing a required data collection function in the necessary location, DP will return a message (lines 16–17). When the first identified resources are stored into **resToExploreNext** array, they are used by the **discover(algoType, resToExploreNext)** function as the resources from which **discover(algoType, resToExploreNext)** will start the traversal of the resource graph, to discover the rest of the necessary resources to satisfy f (line 18). As stated in line 19, the identified resources that provide all of the required functions are stored in the output **discovered** array (line 19). If there are no overlapped resources for any required function in WM, one resource composition is returned. If not, the discovered resources will take part in the Selection Process (SP).

### 4.4. QoR-Driven Resource Selection

#### 4.4.1. Formal Resource Graph Model for i-Compositions

The functions that are necessary to answer a user request define a Workflow Model, WM, such that WM ⊂ FG. Based on the order of the dependent functions that are defined in WM, the identified resources by the discovery process (see Section 4.3) are linked together, forming a Directed Resource Acyclic Graph, DRAG, that is defined as:

**Definition** **4.** *$\mathrm{DRAG}=(\mathrm{DRES},\mathrm{Rel},f_{\mathrm{DRES}},f_{\mathrm{Rel}})$:*

- ***DRES*** *, the set of the discovered static/dynamic resources.*
- ***Rel*** *, the set of relations linking the resources together.*
- *$f_{\mathrm{DRES}}$, the function computing the score of each resource function based on QoR values.*
- *$f_{\mathrm{Rel}}$, the function linking the resources together, and computing their link score based on their I/O similarities.*

The identified resources having the same function, form together a resource group, ${\mathrm{RG}}_{f}$, that is specific to that function, with: ${\mathrm{RG}}_{f}$ = ${⋃}_{i=1}^{m}\left\{{\mathrm{res}}_{\left(f,i\right)}\right\}$, such that m refers to the candidate resources number providing f, and ${\mathrm{res}}_{\left(f,i\right)}$ represents the resource, ${\mathrm{res}}_{i}$, realizing f. A resource composition, RC, that is able to satisfy a user request, is formed by a set of resources, belonging each to a different ${\mathrm{RG}}_{f}$, and such that: **RC** = ${⋃}_{f=1}^{n}\left\{{\mathrm{res}}_{\left(f,i\right)}\right\}$, with n denoting the number of functions defined in WM, and i∈m, where m refers to the number of resources in the relevant ${\mathrm{RG}}_{f}$. During the resource selection process, the matching between the I/O parameters of the linked eligible resources (which align with the required user constraints) is calculated, to form the score of the link relating these resources. This score is computed as: sim(${\mathrm{res}}_{\left(f,i\right)}$, ${\mathrm{res}}_{\left(f^{\prime },j\right)}$) = $\sum_{u=1}^{U}\sum_{v=1}^{V}\mathrm{sim}\left({\mathrm{out}}_{u}^{\mathrm{res}(f,i)},{\mathrm{in}}_{v}^{\mathrm{res}(f^{\prime },j)}\right)$, with:

- ${\mathrm{res}}_{f,i}$, ${\mathrm{res}}_{f^{\prime },j}$, denote resources that belong, respectively, to ${\mathrm{RG}}_{f}$ and ${\mathrm{RG}}_{f^{\prime }}$, where f precedes f’ in WM.
- ${\mathrm{out}}_{u}$, is an output of ${\mathrm{res}}_{f,i}$, and U is the total number of ${\mathrm{res}}_{f,i}$ outputs.
- ${\mathrm{in}}_{v}$, is an input of ${\mathrm{res}}_{f^{\prime },j}$, and V is the total number of ${\mathrm{res}}_{f^{\prime },j}$ inputs.

The score of the matching between an output of a resource and an input of another resource can be calculated by adopting any function for similarity measure between keywords (like Jaccard measure [36] for instance), such that sim(${\mathrm{res}}_{\left(f,i\right)}$, ${\mathrm{res}}_{\left(f^{\prime },j\right)}$) ∈ [0, 1].

Each resource composition (RC) in DRAG has a score, score(RC), with: **score(RC) = Score(RES) + Score(Rel)**, and where:

- **Score(RES)** = $\sum_{f=1}^{n}\mathrm{score}\left({\mathrm{res}}_{\left(f,i\right)}\right)$, is the sum of the scores of the involved resources realizing the required functions, with n is the total number of functions in WM.
- **Score(Rel)** = $\sum \mathrm{sim}({\mathrm{res}}_{\left(f,i\right)}$, ${\mathrm{res}}_{\left(f^{\prime },j\right)})$, is the sum of I/O similarity scores of each 2 eligible linked resources in RC, where: f precedes f’ in WM, and sim(${\mathrm{res}}_{\left(f,i\right)}$, ${\mathrm{res}}_{\left(f^{\prime },j\right)}$) ∈ [0, 1].

Score(RES) and Score(Rel) can be multiplied respectively by $w_{\mathrm{qor}}$ and $w_{\mathrm{io}}$, i.e., weight values that are defined in W included in user-given constraints, C, allowing users to assign them a priority during compositions score calculation.

**Figure 5:**
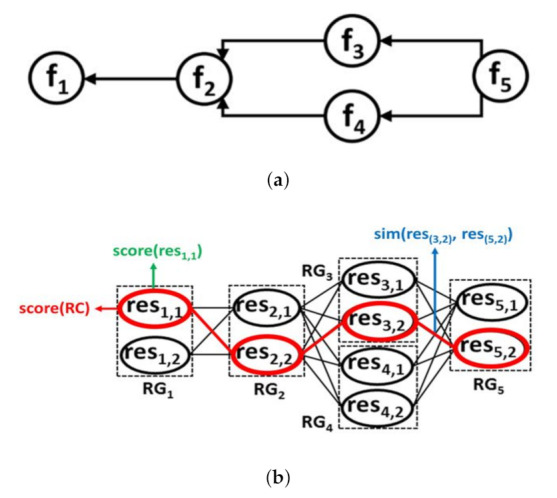
An example of a DRAG showing the scores defined for a possible composition, each of the involved resources and their I/O matching. (**a**) A workflow model example; (**b**) The corresponding DRAG example.

Figure 5 presents a DRAG example that is formed by identified resources. As shown in the figure, the resources discovered by the discovery process are grouped in different resource groups, such that each resource group, RG (represented by a rectangle), holds the resources providing identical function. This is done based on the Workflow Model (WM) which defines the dependencies between the functions required to answer the requested user function $f_{5}$. Each resource in DRAG has a specific score that is calculated based on the quality attributes values related to the necessary function provided by the resource, i.e., ${\mathrm{vf}}_{i}$, and the attribute values related to the resource itself, i.e., ${\mathrm{vres}}_{i}$. The link between every two related resources has a computed similarity measure score (e.g., $\mathrm{sim}({\mathrm{res}}_{\left(3,2\right)}$, ${\mathrm{res}}_{\left(5,2\right)})$ which is relative to the link score between ${\mathrm{res}}_{3,2}$ and ${\mathrm{res}}_{5,2}$). For each possible resource composition (RC) in DRAG that is represented by a path (see for example the resources circled in red in Figure 5) linking one resource included, each, in a different resource group, has an assigned score, score (RC). Next, we present the Selection Strategy Adapter that uses DRAG to generate the necessary resource compositions to satisfy user needs.

#### 4.4.2. Selection Strategy Adapter for i-Compositions

To satisfy different users needs, we define a Selection Strategy Adaptor (SSA) that allows one to generate 3 main types of resource compositions:

1. **Optimal**, denotes the resource compositions with the highest score, score(RC).
2. **Optimistic**, denotes resource compositions having minimally acceptable scores, i.e., computed based on a specific threshold, and formed in satisfactory delays.
3. **Hybrid**, refers to resource compositions that have minimal; acceptable scores, and where the dynamicity aspect of resources is considered, to ensure that, at any time, there is always a resource composition consisting of available resources, providing all the required functions for the user request.

Optionally, each of the above resource compositions types may be followed by other compositions subtypes:

(A) **Trusted**, designates resource compositions that consist only of static resources having Dynamicity = 0.
(B) **Cost-free**, refers to resource compositions composed of resources with no charge of use (i.e., their Cost = 0).
(C) **Efficient**, refers to resource compositions that are formed by resources having a high normalized Usage value (i.e., Usage ⩾ 0.75).
(D) **Effective**, denotes resource compositions that include resources having a high normalized Availability value (i.e., Availability ⩾ 0.75).
(E) **Qualified**, refers to resource compositions that consist of resources, having each, and for a required function, a score(${\mathrm{res}}_{f}$) ⩾ [(n × 0.75) + (m × 0.25)], such that n denotes the number of the QoR attributes (that are to be maximized) related to each resource and its provided required functions (except the Dynamicity attribute), and m refers to the QoR attributes (that are to be minimized), e.g., Cost.
(F) **Reliable**, denotes resource compositions in which Score(Rel) ⩾ (l × 0.75), where l is the number of dependency links that existed in the necessary functions defined in WM.

As shown above, the compositions subtypes are defined according to either: (i) a specific QoR attribute value (e.g., Availability for effective compositions), or (ii) a set of QoR attributes values (e.g., Availability, Cost, and Usage for qualified compositions), or (iii) a minimal Score(Rel) (as for reliable compositions), computed based on “l”, which is the number of dependencies links between the required functions in WM. However, in addition to these constraints, both optimistic and hybrid compositions types should respect other QoR attributes and Score(Rel) values, as presented in Table 1, to ensure having compositions with an acceptable score(RC), and thus, good compositions results.

When end-users request optimal resource compositions subtypes, such as optimal cost-free compositions for instance, which consist of resources with no cost for use, SSA applies a filtering process before computing the highest scores of the possible compositions and retrieving the suitable ones. The filtering process is done according to constraints that are specifically defined for the different resource compositions subtypes as follows:

- Optimal Trusted: denotes resource compositions consisting only of static resources and having the highest compositions scores.
- Optimal Cost-free: refers to resource compositions that include either static or dynamic resources with Cost = 0, and have the highest compositions scores.
- Optimal Efficient: represents resource compositions that are formed by static or dynamic resources having the maximum Usage attribute value among all DRAG resources, and have the highest compositions scores.
- Optimal Effective: denotes resource compositions consisting of static or dynamic resources having the maximum Availability attribute value among all DRAG resources, and having the highest compositions scores.
- Optimal Qualified: refers to resource compositions that are formed by static or dynamic resources with the maximum Score(RES) among all DRAG resources, and have the highest compositions scores.
- Optimal Reliable: represents resource compositions that include static or dynamic resources having the maximum value of Score(Rel), and have the highest compositions scores.

**Figure 6:**
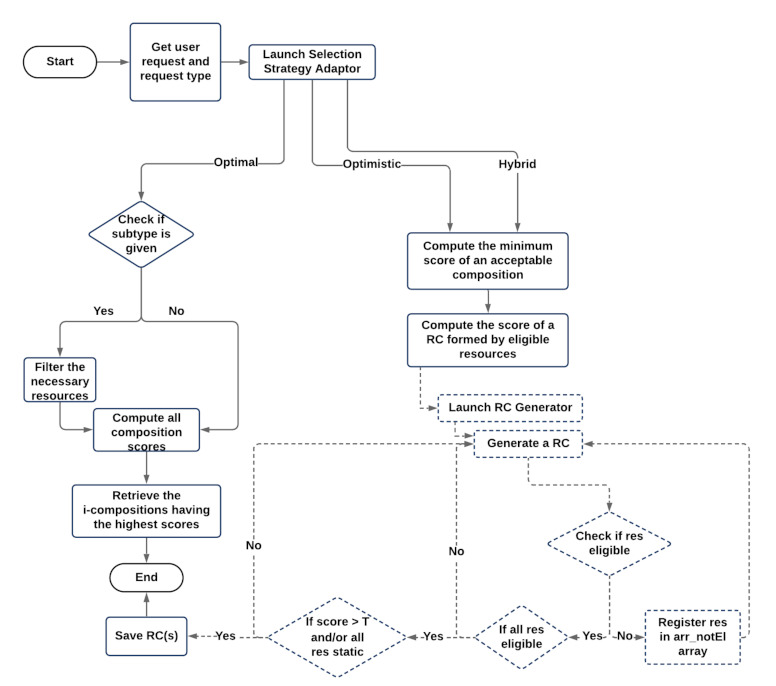
Flowchart of the selection process and its related SSA.

Based on the request type specified by the user, SSA forms i-compositions satisfying his demand. The value of i ($\in N^{*}$) may be defined by the user in his request, r, only for the 2 main resource compositions types: optimal compositions and optimistic compositions. For the hybrid resource compositions, the value of i depends on the dynamicity aspect of the resources contained in DRAG. As such, whenever optimal resource compositions are needed, SSA works on computing all of the possible resource compositions scores (after applying a filtering process if required) to get the i-compositions solutions with the highest compositions scores. Whenever optimistic resource compositions are requested, SSA keeps computing the scores of the possible compositions until reaching the i-compositions having the minimally acceptable scores. If hybrid resource compositions are requested by the user, SSA will generate the compositions having acceptable minimal scores, until having a resource composition that contains only static resources, to ensure that there is always, at any instance of the runtime environment, an available composition realizing user demand. It is to be noted that whenever the user in his request, r, specifies constraints that do not align with the user constraints related to the requested resource compositions subtype, the latter will be considered. In Figure 6, we present the flowchart of the resource selection process along with its relative SSA, used to form the required i-compositions satisfying user request and user request type.

If optimistic or hybrid resource compositions are requested by the user, several steps are applied by SSA:

1. Computing the minimum acceptable score of a suitable composition. A resource composition is considered to be acceptable, if it has a score(RC) that is ⩾a specific computed Threshold, T.
  Whenever resource compositions of optimistic or hybrid types are required without specifying a subtype, T is computed as: T = $\left[\left(n\times \mathrm{Avg}\left(Q_{c}\right)\right)+\left(l\times 0.5\right)\right]\times \left(d/100\right)$, with:
  - **n** denotes the number of functions presented in WM.
  - **Avg($Q_{c}$)** refers to the average value of the QoR normalized constraints that are specified in r (except the Dynamicity attribute). In case $Q_{c}$ are not defined by the user, the average of each QoR is computed based on their maximum values among all DRAG resources. In Figure 6, we present the flowchart of the resource selection process along with its relative SSA, used to form the required i-compositions satisfying user request and user request type.
  - **l**, refers to the number of the links (dependencies) relating to the functions in the Workflow Model. In our work, we assume that, at least, and between any two related resources, there is an I/O similarity match equal to 0.5.
  - **d**, denotes the value of the resource composition acceptance degree (expressed in %) that is specified by the user in his request.
  If the user requires subtype resource compositions, T is calculated as follows: **$T_{\mathrm{subtype}}$** = [(n×Q)+(l×s)]×(d/100), where Q consists of the minimum resources attributes values as defined in Table 1 (except the Dynamicity attribute), and s ∈ [0,1] refers to the minimum value of the I/O matching similarity score between any two related resources in a composition. It is to be noted that s = 0.75 when subtype = reliable, and s = 0.5 for all other compositions specified subtypes.
2. Computing the score of a resource composition that consists of eligible resources. For this matter, SSA uses a generator to retrieve the set of all possible resource compositions without computing their scores. While generating each resource composition, several conditions are applied:
  (i) If a resource in a composition is not eligible (i.e., it does not align with the required user constraints), it will be registered in an array (**arr_notEl**), and another resource composition will be formed.
  (ii) If all of the resources of a composition are eligible, the resource composition score, score(RC), will be computed. When score(RC) ⩾ **T**, the relevant resource composition is saved in an array containing all of the suitable resource compositions, **arr_suitRC**, if not, another possible resource composition will be generated.
  During the analysis of each generated resource composition, if it contains a resource that is already present in **arr_notEl**, another possible composition will be generated. If it is not the case, both conditions (i) and (ii), mentioned above, are applied. We note that whenever optimistic resource compositions are requested by the user, the generator will stop its process when getting i-compositions having a score respecting T. However, when hybrid resource compositions are required, the process of the generator will end when reaching a resource composition whose score respects T, and which consists of static resources only (always available resources). The retrieved resource compositions from SSA are put in **arr_suitRC**.

## 5. Evaluation and Discussion

**Figure 7:**
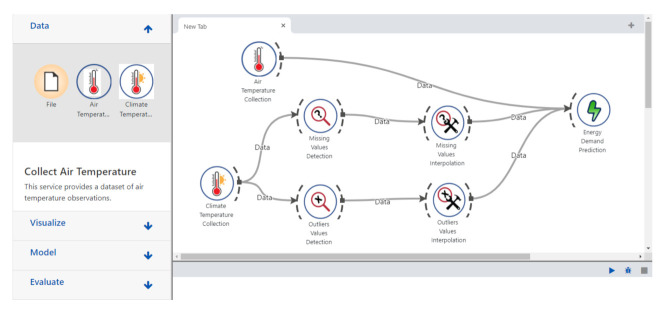
The visual representation obtained in OpenCEMS of the composition example related to our motivating scenario.

**Figure 8:**
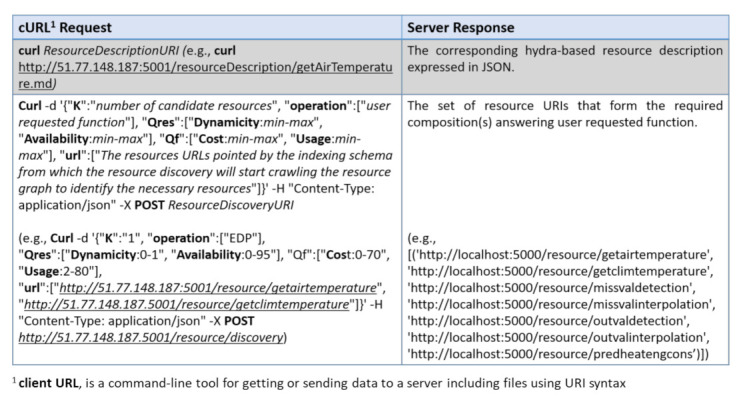
Requests vs Responses in OpenCEMS.

In this section, we first compare our QoR model to existing works that considered services attributes in their service selection solution, and then, we evaluate the performance of the resource discovery and selection in a simulated Web environment provided by OpenCEMS. OpenCEMS offers 2 types of operation: real and simulated. As the real environment is currently being developed with a limited number of resources, in this paper, we evaluated our work in the simulated functioning of OpenCEMS. The evaluation in the real environment will be presented in a dedicated work. However, to prove the feasibility of our approach in the real world, we integrated Hydra-based descriptions for several resources implemented in OpenCEMS used for data collection, data preprocessing, and data energy prediction. Hydra-based descriptions of the implemented resources in the real OpenCEMS environment are available online: https://tinyurl.com/yarlsslp. During the integration, and for each provided function in a resource description, we added an image link to allow composition visualization in OpenCEMS. Based on the user requested function (as EDP which stands for Energy Demand Prediction) and given QoR constraints, OpenCEMS can provide the user with the required visual composition answering his request, as shown in Figure 7. Details about the basic requests sent to the OpenCEMS server with their relative responses are available in Figure 8.

For the resource discovery evaluation, we tested the approach according to the “Location” dimension [27] evaluates the discovery based on Function and Resource dimensions, while varying (1) the number of the needed data collection resources in the required location, (2) the number of locations relative to r, i.e., a non-context aware request ($r^{\mathrm{nca}}$), and (3) the number of required data collection functions provided by resources in the necessary location. As for the selection, the tests were conducted on different DRAG graphs, while varying (1) the number of candidate resources per function, and (2) the number of required functions. The experiments have been conducted by using a Linux Debian (64 bits) virtual machine, having 1 dedicated Intel® Core™ i7-46000 CPU @ 2.10GHz 2.70GHz processor, and 1 GB of RAM. In the results, we present, for each test, the response time (expressed in ms), computed according to 5 sequential executions on average.

### 5.1. Comparison with Existing QoR Models

Our QoR model is designed in a way that it can support any number and type of QoR attributes, as long as these attributes can be added to the resource description. Therefore, we do not consider the number or the type of QoR taken into account when highlighting our contribution with respect to related work [5,13,14,15].

In [5] the performance, availability, and reputation attributes are considered, and simple weighted attribute comparison enables resource selection. Such work does not allow optimistic/hybrid compositions to have acceptable overall service scores, in addition, it ignores I/O matching of the linked services. Also, and due to the missing normalization operations of QoR attributes, the work allows the selection of services having very high values for some attributes and very low values for others, over services with average values for all attributes.

Other work [14], defines constraints such as response time or availability over the whole composition instead of individual services, and use aggregation functions to compute the acceptability of each solution based on weighted services attributes. In contrast, we assign user constraints to individual services and allow weights to be set to the score of the overall service of a composition, as well as to the overall I/O score that is not considered in [14].

We also follow a different approach than [15], as they generate a score based on a weighted sum of utility functions for each service attribute, to select, for a specific task, the service having the highest score. However, in our work, we generate a global composition score and include I/O matching, currently ignored in [15].

The work in [13] selects services based on 4 attributes: Cost, Response Time, Throughput, and Reliability, to form one service composition. Contrary to our work, user QoS constraints are not considered, as user requirements are expressed only in terms of tasks workflows. The approach returns the service composition having the highest fitness value computed based on aggregation functions related, each, to a specific attribute. Apart from not considering I/O matching between the related services, the solution forms the optimal composition without allowing to have other compositions with acceptable scores answering different user needs.

In Table 2, we present the evaluation summary of the aforementioned existing works based on the following service/composition quality-related criteria:

- **QoS Normalization**, indicates if the QoS attributes, that are considered during the service selection process, are normalized.
- **Overall Composition Score**, indicates if an overall score is calculated and assigned to every possible composition.
- **Service Score**, indicates if a score is calculated and assigned to every service.
- **I/O Matching**, indicates whether the Input/Output (I/O) matching of the related services in a service composition is considered during the service selection process.
- **Weights**, indicates if weights can be assigned for each QoS attribute during the service or composition score computation.

In the below table, we used the “+” symbol to express a positive coverage for a criterion, and the “-” symbol to express a lack of criterion coverage.

### 5.2. Resource Discovery Evaluation

The resource discovery process aims to allow the identification of all the resources needed to fulfill the functions required to satisfy user request, r (i.e., we considered that k = 0 in r). The process includes the discovery of the resources that provide the necessary functions for collecting data, while taking into account their locations, and the resources providing the other needed functions (useful for the processing of the collected data) required for r. Our experiments for the resource discovery process have been conducted by using a generated Function Graph, FG, that contained 50 randomly ordered functions. From these functions, we assigned one function for each simulated resource in the generated resource graphs on which we conducted our tests. In the results tests, we show the response time of the discovery process, which corresponds to the time taken to (1) identify the data collection resources using IdS (the Indexing Schema), and (2) to crawl the resource graph based on the identified resources from IdS using the Depth First Search (DFS) algorithm to discover the rest of the necessary resources.

In our tests, we studied the evolution of the resource discovery response time while varying 3 metrics: (1) the number of the needed data collection resources in the required location for user request, (2) the number of locations relative to the user request (a non-context aware request, $r^{\mathrm{nca}}$), and (3) the number of required data collection functions provided by the resources in the required location for the user request. For each of these metrics, we applied different scenarios by generating various resource graphs and distributing the data collection resources to a different number of locations. This is done to see how well our solution can perform according to each metric. For instance, for the metrics related to the number of resources in the required locations, we distributed the resources on 12 locations, as we aimed to test the performance of our solution while increasing the number of resources that are included in the required location for user demand. However, for the second metric, where our objective was to test the performance of our solution while varying the number of required locations necessary for user demand, our resources were distributed on up to 50 locations (instead of 12 locations). Therefore, we needed to have a fixed bigger number of resources in the targeted Web environment (formed by the generated resource graph) to emphasize more on the metric that is being considered.

**Figure 9:**
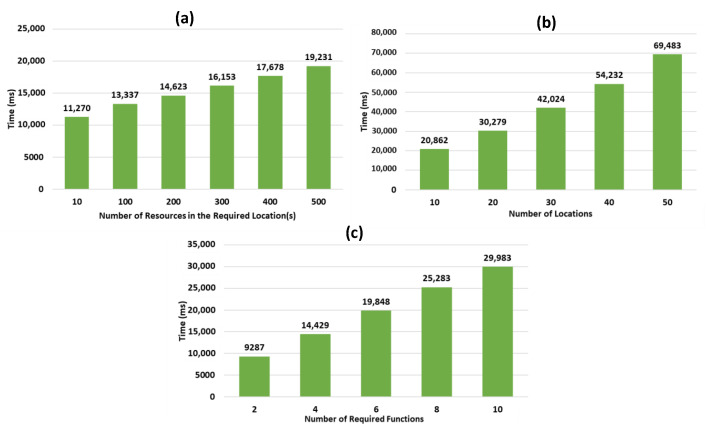
Response time of the resource discovery.

Concerning the first scenario (see Figure 9a), we worked with 2000 resources (1000 static and 1000 dynamic), providing, each, a function from the function graph, and data collection objects were distributed on 12 different locations corresponding to building zones. We explain the response time increase with the number of the required resources to be identified in the necessary locations, and the number of HATEOAS links to be followed from these resources.

Concerning the second scenario (see Figure 9b), we worked with 5 generated graphs containing, each, 4000 resources (2000 static and 2000 dynamic), and up to 50 locations, each of them including 20 resources for data collection. Similarly, we observe here that the cost is due to the number of necessary resources used for collecting data to be discovered along with the HATEOAS link crawling.

Concerning the third scenario (see Figure 9c), we considered 2000 resources (1000 static and 1000 dynamic), among which the resources used for collecting data were distributed on 12 locations, with a varying number of data collection functions necessary to satisfy user requests in the required location. The increase of response time, in this case, is explained by the number of discovered resources that provide additional needed functions. We also observe that in this scenario the impact is more important than in the previous ones.

As shown in Figure 9, the generated graphs in all of the 3 scenarios show a promising and positive linear curve, denoting that the time of the resource discovery process increases linearly with the number of resources in the required location, the number of locations relative to the user request, r, and the number of required data collection functions. This indicates a proportional relation and a constant increase between the different variables used and the response time of our resource discovery process. The results also highlight the important impact of the growing number of locations relative to the user request, on the increase of the resource discovery response time, comparing to the other variables for which the graphs have a smaller slop.

### 5.3. Resource Selection Evaluation

For the selection process evaluation, we conducted several tests by considering two cases: (1) varying the number of candidate resources per a required function (with the Workflow Model, WM, consisting of 5 functions), and (2) varying the number of functions required in WM (here the number of resources per function is fixed to 5). In the tests, we considered that the requested composition type = hybrid. This is done for two main reasons: (a) to focus on how our selection approach performs when considering resources dynamicity forming the compositions, which is one of the key aspects that distinguish our work from existing ones, (b) to the fact that it can cover the case where optimal compositions are requested when all DRAG resources are dynamic and eligible (see the scenario (iii) in the below tests). For each of the 2 cases, we applied several scenarios:

(i) All static resources in DRAG are eligible (match the required user constraints).
(ii) 50% of the static resources in DRAG are eligible.
(iii) All DRAG resources are dynamic and eligible.

**Figure 10:**
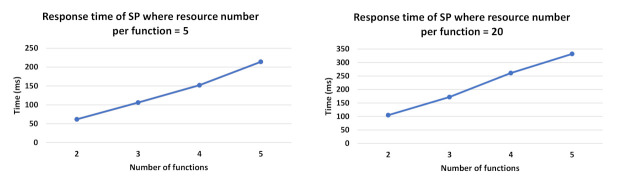
Response time (in ms) of SP while varying resource number per function and the number of required functions.

For (i) and (ii), where DRAG contains static resources, the first generated possible compositions (without score calculation) included dynamic resources, thus, the selection process continued generating compositions until having one that consists on only static resources with an acceptable composition score. In the best case of these two scenarios, static resources are traversed first, and the selection process responds more rapidly. This is shown in the Figure 10 results that present linear curves in the resource selection response time, with a small slope. Such slope slightly increases with the evolution of the number of resources providing the same required function for user request.

In (iii), the selection process computes the score of all the possible resource compositions since the DRAG graph includes dynamic resources that are eligible. The obtained result is close to where the requested subtype = optimal, in which all of the possible resource compositions are calculated. However, in the latter case, i-compositions having the highest scores are retrieved instead.

In the tests, we used a function graph of 50 functions, assigned 2 inputs and 2 outputs for each resource, and specified some user constraints for the Dynamicity, Availability, Cost, and Usage attributes. In our experiments, both static and dynamic resources can participate in the returned resources compositions. We also assumed an I/O similarity score ⩾ 0.5 between the related resources, to allow them to be considered in the possible returned resource compositions having an acceptable minimum score.

**Figure 11:**
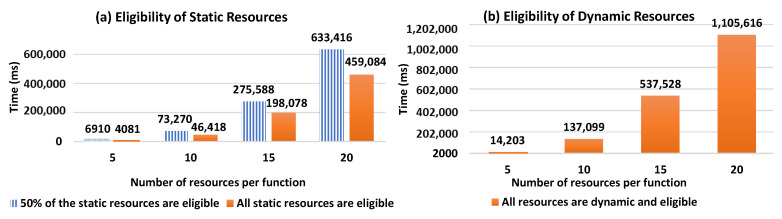
Response time while varying the number of resources.

Figure 11 shows the evolution of the resource selection response time with respect to the number of candidate resources per function. When comparing Figure 11a to Figure 11b, we can observe that the presence of eligible static resources improves response time as it shortens the selection process, which stops when a composition of static resources is found. However, this is not the case when the resources in DRAG are all dynamic and eligible. As such, in Figure 11b, we observe how our solution performs in a context where only dynamic and eligible resources are identified during the resource discovery process to form DRAG. Such a context requires all the compositions to be computed and is more costly in terms of response time, as there will be no composition that will consist of only static resources.

**Figure 12:**
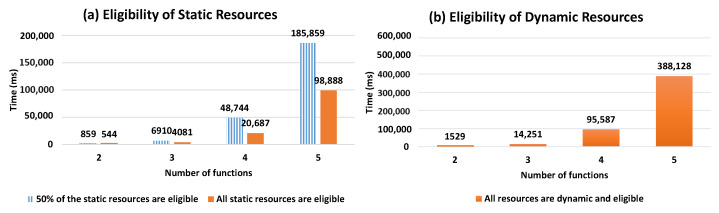
Response time while the varying number of functions.

In Figure 12, we show the evolution of the resource selection response time with respect to the number of the required function necessary to answer user requests (i.e., the number of functions forming the Workflow Model). As shown in Figure 12a, it is expected that the response time will increase together with the number of required functions. Similarly to Figure 11, we can observe early completion of the selection process when static resources are available before generating all possible compositions, comparing to when DRAG includes only dynamic and eligible resources.

As such, in Figure 12b, we can see the significant increase in response time as the DRAG contains dynamic eligible resources, making it necessary to compute all the scores.

Our conducted experiments demonstrate the value of the existence of static eligible resources in DRAG (when type = hybrid) because the selection process stops when forming a composition that consists of static resources with an acceptable score. As such, from the test results, we can observe that whenever the resources in DRAG are all dynamic, our resource selection process will compute all of the scores of the composition (as no composition will consist of static resources), and thus, the response time will be the same as if optimal compositions are required (since all the compositions in such case will also be computed to return the ones having the highest scores). As the dynamicity criteria need to be included in the presence of dynamic resources, we can see that it negatively affects response times with respect to the increase of the number of resources in DRAG, as well as to the increase of the number of required functions in the Workflow Model necessary to answer user demand. The results, however, show that the growing number of resources has a bigger impact on the response time, comparing to the evolution of the number of functions. We note that in case the user desires not to consider the dynamicity aspect of resources while generating the necessary compositions for his demand, he can specify the optimistic composition type in his demand, and thus, the response time of the selection process will depend on the number of the compositions desired by the user in his request.

## 6. Conclusions

In this paper, we present a solution for location-aware resource discovery that considers object location during resource identification, using a 3-dimensional indexing schema. The defined schema facilitates resource discovery in large Web environments connecting numerous resources. We also propose a QoR-driven resource selection approach that relies on a Selection Strategy Adaptor to form i-compositions (with $\in N^{*}$) offering different composition alternatives, and answering different user needs.

We defined optimal, optimistic, and hybrid composition types, as well as trusted, cost-free, efficient, effective, qualified, and reliable composition sub-type, based on various QoR constraints such as Dynamicity, Cost, Availability, or Usage, and data matching (input and output data of related resources), allowing for a better answer to different users needs. Our solution is relevant to Web environments that include both static (always available) and dynamic (frequently disconnected) resources. In addition to the theoretical foundation, we provide experiments and comparisons with existing solutions, that show the advantage and applicability of our solution.

Our experiments show promising good performance for our resource discovery approach, as the response time in all of the conducted scenarios increases linearly with the increased number of the resources in the required locations, the locations in which resources are distributed, and the required functions for the user request. As for our resource selection performance, our tests show that the more dynamic resources are connected to the Web environment, the more the response time increases (whenever the dynamicity of resources is to be considered). This highlights the importance of the existence of static resources. As for the comparisons with existing solutions, they show our coverage for several interesting criteria when computing services and compositions scores, comparing to the other related works.

In the future, we plan to experiment with other setups (e.g., varying simultaneously the number of resources and required functions, consider other compositions types and subtypes) and test the proposed solution in the real Web environment offered by OpenCEMS. We also seek to enhance the selection by removing the non-eligible resources during discovery, and later on, propose an automatic orchestration to execute the formed compositions.

## Figures and Tables

**Table 1:** QoR values and Score(Rel) necessary to form optimistic and hybrid compositions subtypes.

	Resources Dynamicity	Resources Availability	Resources Cost	Resources Usage	Score(Rel)
**Trusted**	0	⩾0.5	⩽0.25	⩾0.5	⩾(l × 0.5)
**Cost-free**	0 or 1	⩾0.5	0	⩾0.5	⩾(l × 0.5)
**Efficient**	0 or 1	⩾0.5	⩽0.25	⩾0.75	⩾(l × 0.5)
**Effective**	0 or 1	⩾0.75	⩽0.25	⩾0.5	⩾(l × 0.5)
**Qualified**	0 or 1	⩾0.75	⩽0.25	⩾0.75	⩾(l × 0.5)
**Reliable**	0 or 1	⩾0.5	⩽0.25	⩾0.5	⩾(l × 0.75)

**Table 2:** Evaluation summary of existing works w.r.t. the service/composition quality-related criteria.

	QoS Normalization	Overall Composition Score	Service Score	I/O Matching	Weights
[5]	-	-	-	-	-
[14]	+	+	-	-	+
[15]	+	-	+	-	+
[13]	+	+	-	-	-

## Appendix A. The Discover(String, Array of String) Function

For each resource that is not yet visited (i.e. not included in **currentId**), the corresponding Hydra description is retrieved using the **getResDesc()** function (line 12). In case the function of the provided resource operation matches a function of **F’**, through calling the **functionMatch()** function (line 14), the algorithm checks if the current analysed resource is a virtual one (i.e., it includes the dynamic resources providing its same function) or if it is a static resource (lines 15 to 20). Whenever the resource is virtual, the ids (URIs) of the dynamic resources that are embedded in the virtual resource description (line 16) are stored with their relative provided function within the **discRes** array. Whenever the resource is static, its id is inserted into the **discRes** array along with its relative provided function (line 21). Once the number of the identified resources providing the current function reaches **k** (lines 18 and 22), the function is pulled out from **F’**. This number is computed by calling the **resFound()** function that is implemented apart. In order to continue discovering other necessary resources, the algorithm process explores the annotated links, which are included in the resources descriptions (lines 24 to 26).

Using the specified algorithm type, **algoType**, the discover function, which is included in Algorithm A1, will traverse the Web resources graph, by starting from the resources stored within the **resToExploreNext** array. The variable **currentId** includes the resource id that is currently being processed. Initially, it corresponds to the the first resource held in the **resToExploreNext** array.

**Algorithm A1:** The Discover(agloType, resToExploreNext) Function
